# Acceptability and Implementation Considerations for 40 Hz Auditory Stimulation Using Nature-Based Soundscapes for Cognitive Health Applications: A Qualitative Exploratory Study

**DOI:** 10.3390/healthcare14040512

**Published:** 2026-02-17

**Authors:** Kiechan Namkung, Kanghyun Lee

**Affiliations:** 1 Techno Design Research Institute, Kookmin University, Seoul 02707, Republic of Korea; 2 AUDIAS Co., Ltd., Chuncheon-si 24329, Republic of Korea; kanghyun@audias.co.kr

**Keywords:** auditory stimulation, soundscape, gamma entrainment, acceptability, implementation outcomes, Theoretical Framework of Acceptability (TFA), qualitative study, intervention feasibility, cognitive health

## Abstract

**Highlights:**

**What are the main findings?**

- Embedding a pure 40 Hz sine wave into nature-based soundscapes yielded a generally acceptable listening experience in adults aged ≥ 40 years (*n* = 11), with mid-to-high comfort and immersion (medians = 5) and low unpleasantness (median = 2) on a session-end appraisal of the intended intervention stimulus (40 Hz–ON).
- While overall preference was moderate (median = 4), perceived artificiality varied widely (range 1–7). In interviews conducted after exposure to both conditions, acceptability depended on perceptual integration: natural blending supported “backgroundable” listening, whereas salient low-frequency rumble or a mechanical/artificial timbre contributed to negative reactions.

**What are the implications of the main findings?**

- For cognitive health-oriented delivery, embedding 40 Hz auditory stimulation in nature-based soundscapes may help mitigate listenability barriers by reducing perceptual salience and supporting a more tolerable listening format.
- Implementation should prioritize conservative defaults and acoustic safeguards (gentle onset, conservative default levels, sensitivity options) and routine-friendly delivery (space-oriented playback options, timers/scheduling), supported by coherence-focused guidance and appropriate disclaimers.

**Abstract:**

Background/Objectives: 40 Hz sensory stimulation is being explored for cognitive health applications, but sustained use may be constrained by the listenability of simple 40 Hz auditory stimuli. We examined user-perceived acceptability and implementation considerations for 40 Hz auditory stimulation delivered by embedding a pure 40 Hz sine wave within nature-based soundscapes. Methods: Eleven adults aged ≥ 40 years in Seoul, Republic of Korea were assigned to waves or forest soundscapes (between-participants) and completed a within-session exposure to two conditions within the assigned set: 40 Hz–OFF (soundscape-only) and 40 Hz–ON (soundscape plus an additively layered 40 Hz sine wave). Each condition comprised seven cycles of 50 s playback and 10 s silence (~7 min) with a 10 min washout. After completing both listening blocks, participants provided brief comparative session-end ratings to aid recall and then completed a semi-structured interview focused on detectability and comparative impressions while blinded to condition identity. Following debriefing about the 40 Hz manipulation, participants completed a session-end 7-point Likert appraisal of the intended intervention stimulus (40 Hz–ON). Interview transcripts were analyzed using thematic analysis and interpreted using the Theoretical Framework of Acceptability and Proctor et al.’s implementation outcomes as sensitizing frameworks. Results: Session-end appraisals suggested that the 40 Hz-integrated soundscape (40 Hz–ON) was generally listenable, with mid-to-high comfort and immersion (medians = 5) and low unpleasantness (median = 2), while perceived artificiality spanned the full scale (range 1–7) and overall preference was moderate (median = 4). Interviews indicated that acceptability was governed by perceptual integration: natural blending supported “backgroundable” listening, whereas salient low-frequency rumble or a mechanical/artificial timbre contributed to negative reactions. Implementation-relevant themes highlighted context fit (bedtime vs. morning routines), low-friction automation (timers/scheduling), and conservative acoustic safeguards (gentle onset and default levels). Conclusions: In a single-session evaluation among adults aged ≥ 40 years, embedding a 40 Hz sine wave within nature-based soundscapes was generally acceptable, with acceptability sensitive to perceptual integration and usage context. This qualitative study does not assess clinical or cognitive efficacy. These findings inform implementation considerations for cognitive health-oriented delivery, including space-oriented playback options, simplified automation, conservative acoustic safeguards, and coherence-supportive user guidance without overclaiming.

## 1. Introduction

Dementia is a major neurocognitive disorder with a rapidly increasing global prevalence and an escalating socioeconomic burden; population aging further amplifies long-term care demands and pressures on healthcare systems [1]. Dementia is a syndrome with heterogeneous etiologies, but Alzheimer’s disease is widely recognized as the most common underlying cause [2]. Against this backdrop, there is growing demand for safe, accessible, and everyday-integrable non-pharmacological (or adjunctive) interventions that may complement the limitations of disease-modifying or symptomatic drug approaches [1,2].

From a neural-oscillation perspective, gamma-band activity has been discussed as a key mechanism supporting information exchange and cognition through coordinated neuronal communication [3]. In Alzheimer’s disease and related cognitive impairment, converging evidence indicates alterations in network activity and oscillatory dynamics, including disruptions that implicate gamma-band function, raising the possibility that gamma dysregulation is linked to cognitive decline and may represent a plausible target for noninvasive neuromodulation [4,5].

In this context, 40 Hz sensory stimulation and entrainment research has expanded rapidly in recent years. Preclinical studies have shown that 40 Hz patterned stimulation can induce gamma-related responses in targeted neural circuits and may modulate Alzheimer’s disease-relevant pathology and neuroinflammatory processes in animal models [6,7,8]. Multisensory paradigms (e.g., combined auditory and visual stimulation) have further been suggested to influence broader brain regions than unimodal stimulation, supporting continued investigation of 40 Hz-based approaches as a platform for probing network- and cellular-level mechanisms linked to cognition [7,8].

Human research on 40 Hz gamma-frequency stimulation is also accumulating. Feasibility and pilot studies in individuals with mild probable Alzheimer’s dementia have indicated that daily at-home 40 Hz sensory stimulation can be deliverable and well-tolerated, while also yielding measurable entrainment-related signals and exploratory outcomes relevant to brain structure and function [9]. In addition, work focusing on auditory stimulation has prospectively assessed the safety and acceptability of 40 Hz amplitude-modulated auditory stimulation delivered via smartphones in healthy older adults [10]. Qualitative research has also begun to examine how older adults with mild cognitive impairment (mild cognitive impairment, MCI) experience and appraise 40 Hz sound- and music-based interventions, explicitly foregrounding user experience, perceived burden, and acceptability-related determinants [11].

Across auditory implementations, two signal-design strategies are commonly used. In amplitude-modulated (AM) stimulation, a carrier sound is modulated in amplitude at 40 Hz, creating a rhythmic 40 Hz envelope that can be delivered via everyday devices (e.g., smartphones) [10]. By contrast, an additive-layering strategy superimposes a pure 40 Hz sine component onto existing audio content (e.g., a soundscape) without modulating the base content. Because additive layering preserves the temporal envelope and spectral–timbre structure of the base soundscape (i.e., it does not impose a 40 Hz modulation on the content itself), it is often selected in design contexts where maintaining perceived naturalism and “backgroundability” of ambient audio is a priority. However, whether this design intent translates into perceived naturalness and acceptability in everyday listening—and under what conditions—remains insufficiently characterized.

Nevertheless, much of the 40 Hz entrainment literature has primarily emphasized either (1) experimental confirmation of changes in neurophysiological, pathological, or cognitive indicators [6,7,8,9] or (2) short-term evaluation centered on tolerability, adverse effects, and adherence [9,10,11]. By contrast, implementation-oriented questions remain comparatively underdeveloped—namely, which delivery channels, everyday contexts, content designs, and interaction patterns shape real-world acceptability and sustained use. Implementation research emphasizes that acceptability should be treated as a distinct, conceptually grounded implementation outcome rather than being reduced to the absence of adverse events [12]. Further, the Theoretical Framework of Acceptability conceptualizes acceptability as a multi-construct concept (e.g., affective attitude, burden, perceived effectiveness, ethicality, intervention coherence, opportunity costs, self-efficacy), underscoring the need to explicate user-perceived mechanisms that determine continued engagement [13].

Soundscapes—defined as the acoustic environment as perceived and/or experienced in context—provide a naturalistic medium for designing auditory interventions at the level of “experience,” rather than as isolated tones [14]. This framing is particularly relevant because simple 40 Hz auditory stimuli are often perceived as unpleasant when presented alone, which constitutes a practical barrier to sustained use [11,15]. To mitigate this barrier, prior approaches have combined 40 Hz stimulation with music or embedded 40 Hz components within musical structures (e.g., “gamma music”), reporting more favorable subjective impressions alongside robust 40 Hz auditory steady-state responses [11,15]. However, music-based delivery can be strongly preference-dependent and may introduce personalization and operational complexity (e.g., genre/track selection, managing repetition fatigue), as reflected in qualitative accounts of user experience and acceptability [11]. In contrast, nature-based soundscapes have been studied as broadly restorative auditory environments, with evidence linking exposure to nature sounds to stress recovery relative to environmental noise and indicating associations between positive soundscape perceptual constructs and health-related outcomes [16,17]. Accordingly, soundscapes may offer a pragmatic pathway to transform 40 Hz auditory stimulation into a more context-compatible and sustainable listening experience for everyday routines [14,16,17].

Importantly, waveform and signal-design choices can shape user experience and may also affect how stimulation is delivered and perceived in practice. We selected an additively layered pure 40 Hz sine component (rather than amplitude modulation of the base content) to preserve the temporal envelope and timbral structure of the soundscape and to prioritize perceived naturalism and backgroundability for everyday use. This choice also enables a simple and auditable intensity control (via relative gain of the 40 Hz layer) without introducing content-dependent modulation artifacts that can increase salience or listening effort. Alternative designs (e.g., AM stimulation, square-wave carriers, or music-based “gamma” content) may offer different entrainment-related properties, but they also impose different perceptual and operational trade-offs; in the present implementation-focused study, we therefore prioritized an ecologically compatible listening format and characterized the conditions under which this design intent is (or is not) realized. Comparative work manipulating auditory stimulus waveforms and listening states has reported that 40 Hz sinusoidal stimulation (relative to square-wave stimulation) can elicit stronger 40 Hz neural responses under specific conditions, highlighting a practical design trade-off space for auditory entrainment implementations [18]. Against this background, the present study adopts an additive 40 Hz sine wave layering strategy embedded in natural soundscapes (rather than AM stimulation), enabling an implementation-oriented examination of how this specific signal-design choice is experienced and what determinants shape real-world acceptability.

For readability, we use several technically accurate expressions interchangeably when referring to the same stimulus format in which a pure 40 Hz sine component is additively layered onto a nature-based soundscape (40 Hz–ON), unless otherwise specified (e.g., “40 Hz auditory stimulation,” “40 Hz–ON stimulus,” “integrated soundscape,” and “additively layered 40 Hz sine wave”). The corresponding soundscape-only contrast condition is referred to as 40 Hz–OFF.

Therefore, this qualitative exploratory study characterizes user-perceived acceptability and implementation considerations for 40 Hz auditory stimulation delivered by integrating a 40 Hz sine wave component into nature-based soundscapes for cognitive health applications. Distinct from prior work focusing on amplitude-modulated auditory stimulation or music-based “gamma” content, we examine a nature-based soundscape medium as a context-flexible delivery format and interpret findings using acceptability and implementation constructs. Importantly, this study does not evaluate clinical efficacy; rather, it aims to inform health-relevant deployment by identifying factors that shape tolerability, perceived burden, and anticipated sustained use, and by translating interview evidence into practical, patient-facing delivery considerations (e.g., low-burden routines, automation supports, and conservative acoustic safeguards).

## 2. Materials and Methods

### 2.1. Study Design and Reporting Framework

**Figure 1:**
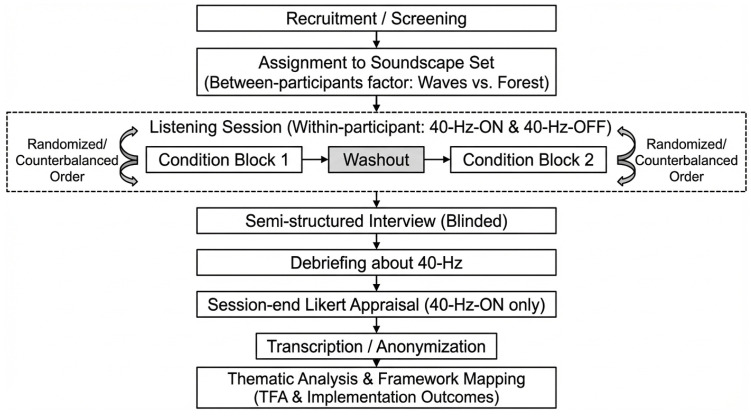
Participants were recruited and screened, assigned to a soundscape set (between-participants), and completed a listening session consisting of two counterbalanced blocks (40 Hz–ON and 40 Hz–OFF) separated by a washout period. After completing both blocks, participants provided brief comparative session-end ratings to support recall and then completed a semi-structured interview while blinded to condition identity. Participants were subsequently debriefed regarding the 40 Hz manipulation and completed a session-end 7-point Likert appraisal focusing on the intended intervention stimulus (40 Hz–ON). Transcripts were anonymized and analyzed using thematic analysis with framework mapping (TFA and implementation outcomes).

We conducted a qualitative exploratory study to characterize user-perceived acceptability and implementation considerations for 40 Hz auditory stimulation delivered by embedding a 40 Hz sine wave component within nature-based soundscapes for cognitive health applications. The overall study procedures were developed and documented in line with the Medical Research Council guidance for complex interventions [19]. Reporting was guided by the Standards for Reporting Qualitative Research (SRQR) [20] and the Consolidated Criteria for Reporting Qualitative Research (COREQ) [21]. Findings were interpreted using the Theoretical Framework of Acceptability (TFA) and Proctor et al.’s implementation outcomes as sensitizing frameworks. Participants were assigned to one soundscape set (waves vs. forest; between-participants) and experienced both stimulus conditions (40 Hz–ON and 40 Hz–OFF) within the assigned set (within-participants). The 40 Hz–OFF condition was included to support contrast-based elicitation of detectability and comparative impressions during the qualitative interview, rather than as a quantitative endpoint for inferential OFF–ON testing. An overview of the study design and delivery workflow is provided in Figure 1.

### 2.2. Ethics

The study was approved by the Institutional Review Board of the investigators’ institution (IRB No. KMU-202509-HR-503). All participants provided written informed consent after receiving information about study purpose, procedures, potential discomfort, and the right to withdraw. Interviews were audio-recorded with consent, transcribed verbatim, and anonymized by removing personally identifying information.

### 2.3. Participants and Recruitment

Participants were recruited in Seoul, Republic of Korea, via online announcements and professional networks. Inclusion criteria were: (1) age ≥ 40 years; (2) no substantial difficulty in everyday listening; and (3) ability to complete the listening session and interview procedures. Hearing was screened using pure-tone audiometry, requiring thresholds ≤ 25 dB HL at key frequencies (e.g., 0.5–4 kHz). Exclusion criteria included neurological disorders (e.g., epilepsy, Parkinson’s disease), ongoing psychiatric treatment or use of psychoactive medication, severe tinnitus, implanted medical devices, pregnancy/breastfeeding, and medications likely to materially affect brain activity.

Eleven adults participated (middle-aged, *n* = 5; older adults, *n* = 6). Seven participants were male and four were female. Participants were assigned anonymized identifiers (P01–P11). Soundscape set assignment (waves vs. forest) was used to diversify content exposure across participants while keeping the 40 Hz comparison within-participants. Condition order by participant is reported in Table 1.

Sample size was selected to support in-depth, implementation-oriented interviewing rather than to achieve thematic saturation. Given the focused study aim (implementation determinants of acceptability for a specific stimulus format) and the use of sensitizing frameworks (TFA and Proctor et al.), we prioritized rich, experience-near accounts across participants with potentially varied listening preferences and contexts. We therefore targeted a small, interview-intensive sample to elicit actionable insights about perceptual acceptability, contextual use scenarios, and practical delivery/UX requirements.

No individuals who were approached declined participation, and no participants withdrew after enrollment.

### 2.4. Auditory Stimuli

#### 2.4.1. Stimulus Sets and Conditions

**Figure 2:**
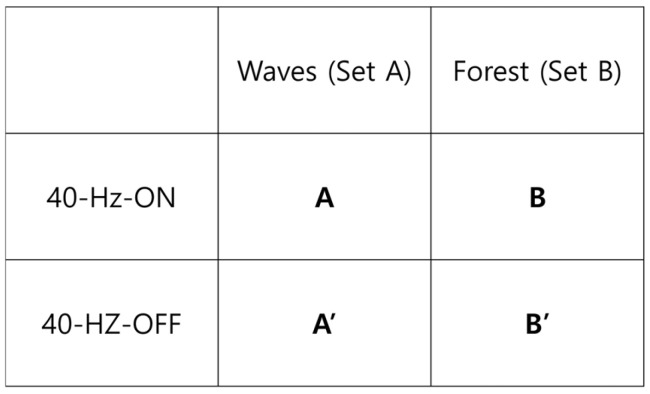
Stimulus structure (2 × 2 schematic). Two soundscape sets (waves and forest) crossed with the absence (40 Hz–OFF; soundscape-only) versus presence (40 Hz–ON) of an additively layered pure 40 Hz sine component.

Two nature-based soundscape content sets were prepared: Set A (waves) and Set B (forest) (Figure 2). Within each set, two final stimulus files were created: 40 Hz–OFF (soundscape-only) and 40 Hz–ON (soundscape plus a 40 Hz component). We use “40 Hz–OFF” and “40 Hz–ON” to denote the absence versus presence of an additively layered 40 Hz sine wave component, respectively (i.e., not amplitude modulation). Primed labels (A′, B′) denote the 40 Hz–OFF stimuli and non-primed labels (A, B) denote the corresponding 40 Hz–ON stimuli. Unless explicitly stated otherwise, preprocessing and mastering procedures were applied identically across OFF and ON files; therefore, “soundscape-only” indicates only the absence of the added 40 Hz component (not the absence of preprocessing).

#### 2.4.2. Pre-Study Calibration for Selecting the 40 Hz Layer Level

Prior to the study sessions, we prepared three candidate versions of the 40 Hz–ON stimulus using different relative 40 Hz layer gain levels within the DAW workflow (Pro Tools, version 2025.6; Avid Technology, Burlington, MA, USA) (low/mid/high; implemented as DAW gain adjustments, i.e., relative level in the digital mixing domain rather than calibrated SPL at the ear). Based on internal pilot listening for study-material preparation, the mid-level setting was selected to balance detectability and listenability, and this setting was used for the final study stimuli.

#### 2.4.3. Production Workflow and Preprocessing

All stimuli were rendered as stereo WAV files (24-bit, 48 kHz) using a consistent editing and mastering workflow across conditions and content sets. To standardize low-frequency content in the base soundscapes and minimize interaction with the added 40 Hz component, the base soundscapes were high-pass filtered at 78 Hz (96 dB/oct) prior to final rendering. This preprocessing was applied identically to both 40 Hz–OFF and 40 Hz–ON soundscape files. For the ON files, the 40 Hz sine wave was added after high-pass filtering so that the 40 Hz component itself was not attenuated.

#### 2.4.4. Loudness Control and Technical Verification

Overall loudness and peak levels were managed to minimize level differences between conditions. To document stimulus levels from the materials actually presented to participants, we conducted post-export verification using waveform statistics extracted from the final rendered files (Table 2). The standalone 40 Hz tone measured −31.3 LUFS integrated loudness (true peak ≈ −24 dBTP). The soundscape-only files measured −18.0 LUFS (waves) and −19.7 LUFS (forest). After additive layering, the final 40 Hz–ON files measured −17.9 LUFS (waves) and −19.4 LUFS (forest), corresponding to small changes in overall loudness relative to OFF (Δ + 0.1 and +0.3 LUFS, respectively). Across all files, no clipping was detected and DC offset remained negligible (Table 2). Key signal-oriented stimulus parameters are consolidated for reproducibility in Appendix A Table A1.

### 2.5. Procedure

Each participant was assigned to one soundscape set (Set A: waves; Set B: forest) and completed a within-session exposure to two conditions within that set: 40 Hz–OFF (soundscape-only) and 40 Hz–ON (soundscape with an additively layered 40 Hz sine wave component). Condition order (OFF → ON vs. ON → OFF) was counterbalanced across participants to reduce order effects (Table 1).

All sessions were conducted in Seoul, Republic of Korea, in a quiet private room within a serviced residence–hotel setting to minimize environmental distractions and support comfortable listening. Participants listened in a seated, relaxed posture. Audio was played from a laptop-based media player (Windows Media Player Legacy; Microsoft Corporation, Redmond, WA, USA) with operating-system (Windows 11; Microsoft Corporation, Redmond, WA, USA) and player volume preset at session start and held constant across conditions; sound pressure level was not individually calibrated. Accordingly, perceived loudness and low-frequency salience may have varied across participants as a function of individual sensitivity and preferred listening levels. Participants listened using wired in-ear earphones (XBA-A2; Sony Corporation, Tokyo, Japan) [22].

Each condition comprised seven cycles of 50 s playback followed by 10 s silence (~7 min per condition). A 10 min washout period was provided between conditions. Immediately after completing both listening blocks, participants provided brief comparative session-end ratings to aid recall for subsequent qualitative probing. Participants then completed a semi-structured interview while still blinded to which block contained the 40 Hz layer. Following the interview, participants were debriefed regarding the 40 Hz manipulation and completed a session-end 7-point Likert appraisal focusing on the intended intervention stimulus (40 Hz–ON).

### 2.6. Qualitative Data Collection and Framework Mapping

Semi-structured interviews were conducted in Korean after completion of the listening session and lasted approximately 30 min per participant. To elicit experience-based descriptions without anchoring to an explicit “40 Hz” label, interviews were conducted while participants remained blinded to condition identity (i.e., which block contained the added 40 Hz layer). Immediately before the interview, participants provided brief comparative session-end ratings (covering both blocks) to aid recall and support condition-referential probing.

The interview guide covered: (i) overall experience and affective response; (ii) perceived discomfort, fatigue, and burden; (iii) detectability and comparative impressions between the two blocks; (iv) perceived value and expected benefits in a cognitive health use case; (v) delivery channel and context preferences (personal listening vs. ambient/space playback; privacy; environmental noise); (vi) routine integration and dosage preferences (timing, frequency, level); and (vii) interaction design requirements (automation, minimized manipulation, and guidance). The guide was mapped a priori to Proctor’s implementation outcomes and TFA constructs to support structured reporting (Table 3).

### 2.7. Quantitative Appraisals

A brief session-end questionnaire was used to provide descriptive context regarding acceptability of the intended intervention stimulus (40 Hz–ON). After the qualitative interview, participants were debriefed about the 40 Hz manipulation and then rated the 40 Hz–ON stimulus on a 7-point Likert scale (1–7) across six items: comfort, immersion, calmness, unpleasantness, perceived artificiality, and overall preference. Given the exploratory intent and small sample size, Likert responses were analyzed descriptively (e.g., median/IQR, range; means/SD additionally reported for transparency), and no inferential testing was performed.

### 2.8. Qualitative Analysis, Rigor, and COREQ Reporting

Interviews were audio-recorded, transcribed verbatim in Korean, and anonymized prior to analysis. We conducted an inductive thematic analysis following Braun and Clarke’s approach [23]. Two researchers (K.N. and K.L.) contributed to the analytic process. K.N. (male; PhD; interaction design background; university faculty member) facilitated the interviews and led primary coding and theme development. K.L. (male; industry practitioner) supported session logistics and participated in analytic discussions to challenge interpretations and refine theme boundaries. Any differences in interpretation were resolved through discussion.

Coding and theme tracking were managed using spreadsheet-based coding matrices, and an audit trail (coding memos and code/theme logs) was maintained to document analytic decisions. Given the exploratory purpose and sample size, we did not aim to establish thematic saturation; themes are presented to reflect a range of perspectives relevant to acceptability and implementation considerations.

Reporting was guided by SRQR and COREQ recommendations; the completed COREQ checklist is provided in Appendix A (Table A2). Illustrative quotations were translated from Korean to English by bilingual researchers and reviewed through discussion to preserve meaning and clarity; no formal back-translation was performed.

## 3. Results

### 3.1. Session-End Likert Appraisal of the Intended Intervention Stimulus (40 Hz–ON; n = 11)

**Figure 3:**
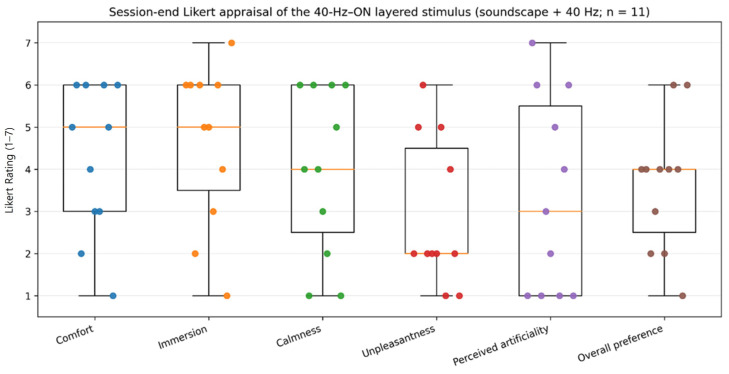
Session-end 7-point Likert appraisals of the intended intervention stimulus (40 Hz–ON; *n* = 11), collected after participants were debriefed about the 40 Hz manipulation. Boxplots show the median and interquartile range (IQR); whiskers extend to 1.5 × IQR. Points represent individual responses (1 = lowest; 7 = highest). No inferential testing was performed.

At the end of the session, participants completed a 7-point Likert appraisal focusing on the intended intervention stimulus, i.e., the 40 Hz–ON layered soundscape (soundscape with an additively layered 40 Hz sine wave component). This questionnaire was administered after the qualitative interview and after debriefing regarding the 40 Hz manipulation, such that ratings were anchored to the identified 40 Hz–ON stimulus. Comfort (median 5, IQR 3–6), immersion (median 5, IQR 3.5–6), and calmness (median 4, IQR 2.5–6) were generally reported at mid-to-high levels. Unpleasantness was relatively low (median 2, IQR 2–4.5). Perceived artificiality showed substantial inter-individual variability (median 3, IQR 1–5.5; range 1–7), and overall preference was moderate (median 4, IQR 2.5–4; range 1–6). Descriptive statistics are summarized in Table 4 and distributions are visualized in Figure 3; no inferential testing was performed.

This session-end questionnaire was used to describe acceptability of the intended intervention stimulus (40 Hz–ON) rather than to compare conditions statistically. In the listening session, the 40 Hz–OFF soundscape was included as a contrast/control to elicit detectability judgments and comparative impressions; brief comparative ratings collected after completing both listening blocks served as recall aids for the blinded interview and were not treated as quantitative endpoints for OFF–ON inferential testing.

### 3.2. Detectability of 40 Hz Inclusion: Variability in Perceived Differences Between 40 Hz–ON and 40 Hz–OFF

During interviews, participants described heterogeneous detectability of differences between the two versions (40 Hz–ON vs. 40 Hz–OFF) within their assigned soundscape set (waves or forest). Responses ranged from “no noticeable difference” to clear discrimination based on perceptual cues. Some participants reported that the two versions felt essentially the same and were uncertain whether any difference existed (e.g., P09). Others described low-frequency sensations such as “vibration” or “bass-like rumble” as a differentiating cue (e.g., P01). Importantly, these descriptors reflect subjective perceptual impressions during listening and should not be interpreted as evidence of 40 Hz neural entrainment, which was not assessed in this qualitative study. A subset characterized one version as having a salient “mechanical/artificial” timbre (e.g., P10), indicating that perceived timbral salience can meaningfully shape preference and willingness to reuse.

### 3.3. Affective Acceptability Hinged on Perceived Naturalness Versus “Mechanical/Artificial” Cues

Building on the perceptual cues described in Section 3.2, affective acceptability hinged on whether the overall listening experience remained naturalistic and restorative versus feeling intrusively “mechanical/artificial.” When the soundscape and added elements were experienced as a coherent ambient texture, participants commonly described the stimulus as comfortable and listenable (e.g., P03). In contrast, acceptability decreased when any component drew attention as an artifact, with some describing discomfort or aversion when the soundscape no longer felt “natural” (e.g., P10). Overall, accounts suggested that acceptability depended less on the abstract concept of “40 Hz” than on perceptual integration—whether the experience could remain backgroundable without persistent attentional capture (e.g., P02).

### 3.4. Burden and Fatigue: Onset Impressions, Level Sensitivity, and Repetition-Related Effort

This subsection addresses burden in terms of early sensory load and repetition-related effort. Overall, participants did not describe severe fatigue as a dominant barrier; however, several noted that the initial moment of listening could feel abrupt or momentarily uncomfortable before becoming more tolerable (e.g., P03; P05). Episodes of irritation were often linked to onset impressions and to recurring sound elements that periodically re-captured attention (e.g., P07). These observations point to practical design considerations for reducing early burden, such as gentle onset handling (e.g., fade-in), conservative default levels, and repetition fatigue management.

### 3.5. Backgroundability as a Sustainability Facilitator

Distinct from the early sensory load described in Section 3.4, “backgroundability” captured sustained listening with low attentional engagement over time. Some participants reported that the audio became background-like within 1–2 min (e.g., P07), which aligned with comfort and anticipated sustainability. In contrast, when listeners remained vigilant or continued to “monitor” the sound for potential layered elements, the experience did not fade and could become effortful (e.g., P10; P03). This pattern suggests that supporting background listening—by minimizing attention-grabbing repetitions and reducing salient “mechanical/artificial” cues—may facilitate routine integration and real-world feasibility.

### 3.6. Context Fit: Bedtime/Relaxation Versus Morning/Active Routines

Participants proposed bedtime relaxation and morning refreshment/walking contexts as plausible use scenarios. This theme aligns primarily with TFA constructs of opportunity costs and self-efficacy, because context-matched routines can lower the time/effort required to start listening and increase confidence in integrating the activity into everyday schedules. Bedtime listening was associated with relaxation and reduced intrusive thoughts, facilitating drowsiness and perceived readiness for sleep (e.g., P01). Morning contexts were linked to refreshment and routine activation (e.g., P03). Some highlighted walking as a feasible setting that supports low-attention listening (e.g., P02). These patterns support routine-based presets (e.g., “Sleep routine,” “Morning routine”) that match context and reduce decision-making burden.

### 3.7. Delivery and UX Preferences: Ambient Playback and Low-Friction Automation

While a minority preferred earphones for personal control, many participants favored ambient speaker playback as more natural and less effortful than wearing earphones (e.g., P05). Participants also expressed a preference for low-friction automation, such as timer-based scheduled playback (“set-and-forget”) and an easily accessible stop function (e.g., P11). In TFA terms, these preferences reflect burden and opportunity costs (reduced hassle and decision effort) and support self-efficacy by making initiation and stopping straightforward, which may facilitate sustained use (see Table 5). Continued use was threatened when listening felt mandatory or externally imposed (e.g., P07), underscoring the importance of autonomy-supportive UX and user-controlled scheduling.

### 3.8. Perceived Value and Intervention Coherence: Cognitive Health Framing and Guidance Needs

Willingness to use increased when the intervention was framed as potentially beneficial for cognitive health (e.g., memory maintenance) (e.g., P01), highlighting perceived effectiveness as a strong acceptability driver. However, participants also indicated gaps in intervention coherence: some could not clearly articulate what differed between versions or how the stimulus might work, despite describing the experience as tolerable (e.g., P03). This pattern highlights intervention coherence as a prerequisite for perceived effectiveness, and suggests that clearer explanations can also support self-efficacy (knowing how/when to use the stimulus) while reducing opportunity costs associated with uncertainty and repeated trial-and-error (see Table 5). In addition, reassurance regarding safety perceptions (e.g., concerns related to tinnitus/ear ringing) appeared important for ethical acceptability (e.g., P11). Accordingly, implementation should provide plain-language explanations and expectation management without overclaiming, accompanied by appropriate disclaimers. A structured mapping of interview-derived themes to implementation outcomes and TFA constructs is provided in Table 5.

## 4. Discussion

### 4.1. Summary of Findings

This qualitative exploratory study examined acceptability and implementation considerations for 40 Hz auditory stimulation delivered by embedding an additively layered 40 Hz sine wave component within nature-based soundscapes for cognitive health applications. Rather than testing clinical efficacy, our aim was implementation-first: to identify determinants of tolerability, perceived burden, and anticipated sustained use, and to translate user evidence into patient-facing delivery requirements (Table 6).

Across participants aged ≥ 40 years, the session-end appraisal of the intended intervention stimulus (40 Hz–ON) suggested that the integrated soundscape was generally listenable, with moderate-to-high comfort and immersion and relatively low unpleasantness, while perceived artificiality varied widely across individuals. Interview findings clarified the practical significance of this heterogeneity: acceptability was shaped less by the abstract concept of “40 Hz” and more by perceptual integration—whether the added component blended into the soundscape or became salient as low-frequency “vibration/rumble” or a “mechanical/artificial” timbre. When salience was low and the audio became “backgroundable,” participants anticipated greater sustainability; when salience was high, aversion and rejection emerged for a subset.

Taken together, the findings support a cautious, implementation-oriented interpretation: soundscape embedding may be a viable delivery format to improve the listenability of 40 Hz auditory stimulation, but real-world uptake is likely to depend on conservative acoustic design and context-sensitive implementation rather than on a one-size-fits-all approach.

### 4.2. Implementation Implications for Scalable Delivery and UX

From a healthcare implementation perspective, cognitive health applications are more likely to be adopted when an intervention is perceived as tolerable, low-burden, and easy to integrate into everyday routines without requiring substantial effort or technical calibration. Participant feedback in this study suggests several implementation and UX considerations (Table 6).

Delivery modality. Many participants described earphone-based listening as effortful, whereas ambient speaker playback was perceived as more natural and potentially more sustainable. This suggests that future deployment may benefit from supporting space-oriented listening (e.g., bedrooms, living rooms, relaxation areas) alongside personal listening options for privacy and control.

Interaction burden. Anticipated sustained use depended strongly on minimizing interaction demands. Participants favored low-friction controls such as one-tap start, scheduled or timer-based playback (“set-and-forget”), and an easily accessible stop function, indicating that simplified interaction may reduce routine friction and support adherence.

Autonomy and optionality. Several accounts indicated that continued use could be threatened when listening felt obligatory. Accordingly, autonomy-supportive design—framing use as optional and user-controlled, and enabling frictionless pausing or discontinuation—may help sustain engagement in everyday settings.

Acoustic acceptability safeguards. A subset reported aversion when the experience was abrupt at onset or when low-frequency rumble or a mechanical/artificial timbre became salient. These accounts suggest that conservative, user-centered safeguards may help support tolerability, such as gentle onset handling (fade-in), sensitivity-oriented alternatives (e.g., lower-intensity mixes), and other practical measures that reduce discomfort. These safeguards are design strategies to support user acceptability rather than clinical safety claims.

Level handling and guidance. Consistent with participants’ emphasis on low-burden use, implementations may benefit from conservative defaults and simple, user-facing guidance rather than calibration-heavy protocols. Brief comfort-oriented prompts and straightforward level adjustment options may support sustained use across heterogeneous listening sensitivities.

Coherence-focused guidance. Willingness to engage appeared to increase when the audio was framed in relation to cognitive health goals; however, some participants were unable to clearly articulate what differed between versions or how the stimulus might work. This indicates that future delivery materials may benefit from brief, plain-language explanations and expectation setting that avoid overclaiming while improving intervention coherence.

Real-world deployment scenarios and comparative testbeds. These implementation determinants could be operationalized via a mobile application delivery layer offering context-matched presets, scheduled playback, and an easily accessible stop/snooze function to preserve autonomy. Given heterogeneous low-frequency sensitivity, app-based delivery could provide conservative defaults, gradual ramping (fade-in), and simple, bounded personalization (e.g., “more subtle/more salient”), alongside plain-language guidance and non-overclaiming expectation setting. As a systems-oriented next step, future implementation pilots could also consider alternative signals (e.g., amplitude-modulated variants or different carriers) as comparative testbeds while holding the delivery UX constant, to isolate perceptual acceptability trade-offs without implying efficacy.

Although the present study does not evaluate efficacy, the acceptability findings can inform the design of future efficacy or neurophysiological studies by reducing avoidable barriers that confound adherence and exposure. Specifically, future trials may benefit from (i) personalization options or stratified protocols to accommodate heterogeneous sensitivity to low-frequency salience, (ii) predefined intensity/level bounds with conservative defaults and gradual ramping, and (iii) context-matched delivery (e.g., bedtime or background ambient playback) supported by low-friction automation and clear, non-overclaiming guidance. These design choices can help standardize “usable” exposure while maintaining ecological validity, without implying causal effects from the present qualitative data.

### 4.3. Limitations

Several limitations should be considered. First, this qualitative exploratory study was not designed to evaluate clinical efficacy or to draw causal conclusions about health outcomes; rather, it focused on user-perceived acceptability and implementation considerations for cognitive health applications. Second, the sample size was small and recruitment was geographically localized, which may limit transferability to other populations and settings. To support transferability judgments, we provide detailed contextual information about the recruitment setting, participant characteristics, listening procedures, and stimulus parameters, along with verbatim quotes that illustrate variability in perceptions. Nonetheless, implementation determinants may differ across cultural contexts, languages, health-status profiles, and real-world listening environments; future work should test transferability in more diverse regions and in intended end-user groups (e.g., older adults with MCI) using repeated-use and field deployment designs. In addition, the session-end 7-point Likert appraisals were collected after debriefing about the 40 Hz manipulation, and thus may be susceptible to expectation effects or demand characteristics. Accordingly, these ratings are interpreted descriptively as reflective self-reports rather than as evidence of perceived effectiveness, particularly given that perceived value and effectiveness were discussed during the interviews.

Third, exposure occurred in a single, controlled listening session; acceptability and routinization may differ with repeated use, longer follow-up, and real-world environmental variability. Repeated exposure could yield both beneficial habituation and adverse repetition fatigue: some listeners may acclimate over time such that initial salience or discomfort diminishes, whereas others may become increasingly bothered by recurring elements or timbral cues as they repeat across sessions. Future longitudinal work should therefore model both trajectories (habituation vs. fatigue) and identify design parameters (e.g., onset handling, variability/rotation of content) that mitigate repetition-related burden while preserving backgroundability. Future work should also assess within-participant test–retest reliability of key acceptability indicators under standardized playback and calibration procedures. Finally, playback volume was preset rather than individually calibrated, and the relative level of the added 40 Hz layer was finalized via internal pilot listening (i.e., DAW gain selection rather than calibrated SPL at the ear). As a result, individual differences in low-frequency sensitivity and auditory comfort thresholds may have influenced perceived acceptability, including the salience of “vibration/rumble” or “mechanical/artificial” timbral cues, thereby contributing to variability in responses. Importantly, such “vibration/rumble” reports are interpreted here as perceptual acceptability cues rather than as evidence of 40 Hz neural entrainment, because this qualitative study did not include neurophysiological measures. Future work should examine these considerations in larger and more diverse samples using repeated-use protocols and field-based deployment, and should consider participant-specific (or adaptive) level setting, standardized calibration procedures, and conservative default levels with gradual ramping to reduce variability attributable to individual sensitivity.

## 5. Conclusions

This qualitative exploratory study suggests that integrating an additively layered 40 Hz sine wave into nature-based soundscapes can be acceptable as an everyday listening experience, although responses are heterogeneous and sensitive to timbral salience and usage context. Session-end appraisals of the intended intervention stimulus (40 Hz–ON) indicated mid-to-high comfort and immersion with a low median unpleasantness; however, interviews showed that perceived low-frequency “vibration/rumble” and “mechanical/artificial” timbral cues could become decisive drivers of aversion for a subset, whereas “backgroundable” naturalistic listening supported anticipated sustainability.

From an implementation perspective, scalable uptake is likely to depend on space-oriented ambient playback options, ultra-low-friction automation and control, acoustic acceptability safeguards (e.g., fade-in and conservative default levels), and coherence-supportive user-facing guidance with evidence-aligned framing and appropriate expectation setting (Table 6). Importantly, these implications pertain to acceptability, feasibility, and implementation considerations of the delivery format for cognitive health applications, not to clinical or cognitive efficacy. Efficacy claims require appropriately powered trials with clinical and/or neurocognitive outcomes and, where relevant, neurophysiological assessment. Future studies should evaluate these implementation refinements in larger and more diverse samples, including target populations such as older adults with MCI. Longitudinal field trials will be important to examine sustained use, routine integration, and preference drift over time, alongside downstream neurophysiological or clinical outcomes assessed in appropriately powered designs.

## Figures and Tables

**Table 1:** Participant characteristics assigned soundscape set, and within-session condition sequence (anonymized). Set A = waves; Set B = forest. Within each set, ON denotes the soundscape with an additively layered pure 40 Hz sine component, and OFF denotes the soundscape-only version. Coding: A = waves (ON), A′ = waves (OFF); B = forest (ON), B′ = forest (OFF). Each participant was assigned to one soundscape set (between participants) and completed both OFF and ON within that set (within participants); the OFF/ON sequence (Condition 1 → Condition 2) was counterbalanced. The OFF condition served as a contrast/control to elicit detectability judgments and comparative impressions in interviews, not as a quantitative endpoint. Age-group labels reflect recruitment stratification (middle-aged: 40–49 years; older adults: ≥60 years).

No	Gender	Age	Age Group	Assigned Soundscape Set	Condition 1	Condition 2
P01	Male	46	Middle-aged	Set A (Waves)	A′	A
P02	Male	45	Middle-aged	Set B (Forest)	B	B′
P03	Male	77	Older adult	Set A (Waves)	A′	A
P04	Male	77	Older adult	Set A (Waves)	A	A′
P05	Female	78	Older adult	Set A (Waves)	A′	A
P06	Female	40	Middle-aged	Set A (Waves)	A	A′
P07	Female	67	Older adult	Set B (Forest)	B′	B
P08	Male	44	Middle-aged	Set B (Forest)	B′	B
P09	Male	48	Middle-aged	Set A (Waves)	A	A′
P10	Female	69	Older adult	Set B (Forest)	B′	B
P11	Male	70	Older adult	Set B (Forest)	B	B′

**Table 2:** Post-export stimulus level verification and technical quality control. Waveform statistics were extracted from the final rendered WAV files to document integrated loudness (LUFS), true-peak level (dBTP), clipping, DC offset, and loudness range (LRA). ON indicates additive inclusion of a pure 40 Hz sine component; OFF indicates the corresponding soundscape-only file. Overall loudness changes after layering were small (Waves: Δ + 0.1 LUFS; Forest: Δ + 0.3 LUFS, ON relative to OFF).

Stimulus (Final Rendered File)	Condition	Integrated Loudness (LUFS)	True Peak (dBTP)	Clipped Samples	DC Offset (%)	LRA (LU)
40 Hz tone (standalone reference)	—	−31.3	−23.97	0	+0.000	0.0
Waves soundscape	OFF (A′)	−18.0	−6.36	0	−0.001	9.7
Forest soundscape	OFF (B′)	−19.7	−8.57	0	+0.000	5.1
Waves soundscape + 40 Hz	ON (A)	−17.9	−6.28	0	−0.001	8.9
Forest soundscape + 40 Hz	ON (B)	−19.4	−8.65	0	+0.000	4.7

**Table 3:** A priori mapping of interview domains to implementation outcomes and TFA constructs. Domains were mapped in advance to Proctor et al.’s implementation outcomes (e.g., acceptability, feasibility, appropriateness) and TFA constructs to support structured qualitative analysis and reporting.

Interview Domain	Implementation Outcome	TFA Construct	Example Prompts (Semi-Structured)	Planned Linkage in Results
Overall impression and willingness to reuse	Acceptability	Affective attitude	“How was the overall experience?” “Would you choose to use this again?” “What did you like/dislike most?”	Emotional drivers of acceptability; preference patterns
Discomfort, fatigue, and perceived burden	Acceptability/Feasibility	Burden	“Was anything uncomfortable or tiring?” “What made it feel effortful?” “How long could you tolerate this per session?”	Barriers to sustained use; fatigue-management needs
Understanding and sense-making of the intervention	Appropriateness	Intervention coherence	“What do you think you were listening to?” “How do you think it works?” “Did the experience make sense to you?”	Guidance needs; labeling/framing; coherence gaps
Expected benefits and perceived value	Acceptability	Perceived effectiveness	“What changes did you notice, if any?” “What benefits would you expect with repeated use?”	Value propositions; perceived mechanisms; outcome expectations
Concerns, ethical fit, and safety perceptions	Acceptability	Ethicality	“Did anything feel concerning or unacceptable?” “Any worries about safety or unintended effects?”	Concerns and mitigation strategies; ethical acceptability
Trade-offs and opportunity costs	Acceptability	Opportunity costs	“What would you have to give up to use this regularly?” “Would this compete with other routines?”	Routine integration; adherence risks; scheduling constraints
Confidence in independent use	Feasibility	Self-efficacy	“Could you use this on your own without help?” “What would make it easier to start/continue?”	UX simplification; onboarding; support features
Delivery channel and context preferences	Feasibility/ Appropriateness	Cross-cutting (contextual)	“Would you prefer earphones/headphones or ambient/space playback?” “Where and when would you use it?” “How does privacy or background noise matter?”	Implementation scenarios (personal vs. ambient); context constraints

Notes. 1. Implementation outcomes categories are informed by Proctor et al. (acceptability, feasibility, appropriateness) [12]. 2. TFA constructs are informed by Sekhon et al. [13]. 3. “Contextual (cross-cutting)” is used for domains capturing implementation context rather than a single TFA construct.

**Table 4:** Descriptive statistics of 7-point Likert ratings for the intended intervention stimulus (40 Hz–ON; *n* = 11), administered after debriefing about the 40 Hz manipulation.

Item	Median (IQR)	Range	Mean ± SD
Comfort	5 (3–6)	1–6	4.27 ± 1.79
Immersion	5 (3.5–6)	1–7	4.64 ± 1.91
Calmness	4 (2.5–6)	1–6	4.00 ± 2.00
Unpleasantness	2 (2–4.5)	1–6	2.91 ± 1.76
Perceived artificiality	3 (1–5.5)	1–7	3.36 ± 2.34
Overall preference	4 (2.5–4)	1–6	3.64 ± 1.57

Notes. Items were rated on a 7-point scale (1–7). Higher scores indicate higher levels of each construct (e.g., higher unpleasantness indicates greater unpleasantness). Given the ordinal nature of Likert items and the small sample size, results are reported descriptively (median/IQR/range; mean/SD provided for reference). IQRs were computed as the 25th–75th percentiles using standard percentile estimation with linear interpolation.

**Table 5:** Interview-derived themes mapped to implementation outcomes (Proctor et al.) and Theoretical Framework of Acceptability (TFA) constructs. Themes were developed via thematic analysis and subsequently interpreted using Proctor’s implementation outcomes framework (e.g., acceptability, feasibility, appropriateness) and TFA constructs (e.g., affective attitude, burden, intervention coherence, perceived effectiveness, ethicality, opportunity costs, self-efficacy) as sensitizing frameworks. Representative quotes are provided using anonymized participant identifiers (P01–P11).

Results Subsection/Theme	Implementation Outcome (Proctor)	TFA Construct(s)	What This Theme Captures (Summary)	Representative Quotes (English Translation; Anonymized IDs)
3.2 Detectability of 40 Hz inclusion: variability in perceived differences (ON vs. OFF)	Acceptability; Appropriateness	Intervention coherence; Affective attitude	Participants varied from “no discernible difference” to clear discrimination based on low-frequency “vibration/rumble” or “mechanical/artificial” timbral cues; this variability shaped preference and willingness to reuse.	P09: “I’m not sure—weren’t they the same sound? I didn’t really think there was a big difference while listening.” P01: “The basic wave sound was the same, but in the second one I felt something like a bass layer underneath—like a vibration.” P10: “There was definitely a difference. The first sounded like natural birds and felt refreshing, but the second had a sharp mechanical sound and felt artificial, so I didn’t like it.”
3.3 Affective acceptability hinged on perceived naturalness vs. mechanical/artificial cues	Acceptability	Affective attitude; Ethicality (sensory aversion)	“40 Hz” as a concept was less salient than the perceived texture: naturalistic soundscapes supported positive affect, while salient artificial/mechanical cues rapidly reduced acceptability.	P03: “Both sounded natural—like sounds you could hear in nature—so I felt comfortable, like resting.” P02: “The first felt somewhat artificial… and some repeating elements were irritating.” P10: “The first felt comfortable; the second was not comfortable at all.”
3.4 Burden and fatigue: onset impressions, level sensitivity, repetition-related effort	Acceptability; Feasibility	Burden	Burden was not generally high, but several participants described the start as loud/abrupt or initially uncomfortable, then becoming tolerable—implying design needs (gentle onset/fade-in, conservative defaults).	P03: “At first the sound felt loud and startled me, but it became okay over time.” P05: “The second one was a bit uncomfortable only at the beginning; as it went on it became fine.” P07: “Because a loud sound came in suddenly at the beginning, it felt a bit strong.”
3.5 “Backgroundability” as a sustainability facilitator	Feasibility; Acceptability	Burden; Opportunity costs	For some, the audio “faded into the background” after 1–2 min (supporting sustainability). For others, it remained foregrounded and attention-demanding, making listening effortful.	P07: “After about 1–2 min, it started to feel like background.” P10: “It didn’t fade into the background; since it kept playing, I listened attentively.” P03: “It didn’t feel like background… I kept thinking about what sounds were mixed in and where they were recorded.”
3.6 Context fit: bedtime/relaxation vs. morning/active routines	Feasibility; Appropriateness	Opportunity costs; Affective attitude	Acceptability was strongly context-dependent. Bedtime/relaxation contexts were linked to calming/rumination reduction, while morning/walking contexts were linked to refreshment and routine activation.	P01: “Before sleep… it made me feel calmer, reduced intrusive thoughts, and made me drowsy.” P03: “It would be good in the morning after waking up… nature sounds feel better earlier in the day.” P02: “It would be good to listen to while walking… ideally so it’s heard naturally, almost unconsciously.”
3.7 Delivery and UX preferences: ambient playback and low-friction automation	Feasibility	Self-efficacy; Burden	Many preferred low-friction delivery (speaker/ambient, radio-like passivity) and automation (scheduled playback). Continued use was threatened if listening felt forced or effortful.	P05: “Through a Bluetooth speaker… it’s easier than having to wear something in the ear.” P11: “The easiest would be scheduled, automatic playback.” P07: “If it feels forced, I would be less likely to keep using it.” P04: “A radio-like way where it plays automatically without much interaction would be most convenient.”
3.8 Perceived value and intervention coherence: cognitive health framing and guidance needs	Acceptability; Appropriateness	Perceived effectiveness; Intervention coherence; Ethicality	Motivation increased markedly when framed as beneficial for cognitive health; however, some participants could not articulate differences/mechanisms, indicating need for plain-language guidance and expectation management. Safety reassurance mattered (e.g., tinnitus concerns).	P01: “Of course. If it helps health, I’ll do anything—I would seek it out and listen.” P03: “I couldn’t tell exactly what the difference was, but there wasn’t anything particularly uncomfortable.” P11: “I didn’t feel any worsening or change in tinnitus or ear ringing while listening.”

Notes. 1. Quotes were translated from Korean to English for reporting; minor edits were made for readability while preserving meaning. 2. “Contextual fit” items are mapped primarily to feasibility/appropriateness and opportunity costs because they specify real-world usage constraints rather than a single TFA construct. 3. Quotes were selected to represent the range of views observed across participants and are not intended to indicate frequency.

**Table 6:** Implementation and UX considerations suggested by user feedback. The table summarizes design considerations implied by participant accounts to support tolerability and low-burden routinization. These considerations are intended to inform future prototyping and field deployment and do not imply clinical efficacy or constitute clinical guidance.

Domain	Actionable Requirement (What to Implement)	Operational Specification (UX/Deployment)
Delivery modality	Support ambient/space playback in addition to personal listening	Provide a “Speaker/Ambient mode” (e.g., living room/bedroom/relaxation area) alongside an earphone/headphone mode; include brief guidance on when each mode may be preferable (privacy vs. effort-free use).
Interaction burden	Reduce manipulation via one-tap start, scheduled/timer playback, and quick stop	Consider implementing: (i) one-tap start of the last-used routine, (ii) scheduled playback and/or a timer (“set-and-forget”), and (iii) an always-accessible quick stop; keep controls sparse (start/stop, level, schedule).
Autonomy and optionality	Keep routines optional rather than mandatory	Enable skip/pause/discontinue without penalties; frame routines as user-chosen presets rather than daily obligations; avoid messaging that implies “must listen every day.”
Acoustic acceptability safeguards	Use gentle onset handling and sensitivity-oriented options	Use a default fade-in at session start; adopt conservative default levels; provide simple volume guidance; offer lower-intensity or alternative mixes for users sensitive to low-frequency rumble or “mechanical/artificial” timbre.
Level handling and guidance	Prefer conservative defaults and simple guidance rather than calibration-heavy protocols	Set a comfortable default playback level; include a short comfort prompt (e.g., “keep at a comfortable level; pause if uncomfortable”); optionally add a brief first-use “comfort check.”
Coherence-focused guidance	Provide plain-language explanation with non-exaggerated framing (and appropriate statements as applicable)	Add a concise “What this is/What to expect” description; avoid efficacy claims; include expectation-setting language and any required statements consistent with the intended use context (e.g., wellness-oriented positioning).

## Data Availability

The data are not publicly available due to ethical and privacy restrictions related to qualitative interview transcripts. De-identified data may be available from the corresponding author upon reasonable request and with Institutional Review Board approval.

## Abbreviations

The following abbreviations are used in this manuscript:

TFA	Theoretical Framework of Acceptability
LUFS	Loudness Units relative to Full Scale
dBTP	decibels true peak
LRA	Loudness Range
HPF	High-pass Filter

## Appendix A

### Appendix A.1. Stimulus Signal-Oriented Parameters

To improve reproducibility of the stimulus design, we consolidate key signal-oriented parameters of the study stimuli (soundscape-only vs. soundscape plus additively layered 40 Hz sine component) in Table A1, including file format, preprocessing (HPF), additive-layering details, post-export level verification, and the within-session exposure structure.

**Table A1:** Consolidated signal-oriented parameters of the study stimuli (soundscape-only vs. soundscape plus additively layered 40 Hz sine component).

Category	Parameter	Value/Setting	Notes
Content	Soundscape sets	Set A: Waves; Set B: Forest	Two content sets (Figure 2).
Conditions	OFF vs. ON	40 Hz–OFF: soundscape-only; 40-Hz–ON: soundscape + additively layered pure 40-Hz sine	Additive layering (not amplitude modulation).
Added component	Frequency	40 Hz	Pure sine component.
	Waveform	Sinusoid	Added after base-soundscape HPF
Mixing/level	40 Hz layer level selection	Three candidate DAW gain settings (low/mid/high); mid selected by internal pilot listening	Relative gain in mixing domain; not calibrated SPL at the ear.
File format	Render Format	Stereo WAV, 24-bit, 48 kHz	Identical render format for all files.
Base preprocessing	High-pass filter (HPF) on base soundscape	78 Hz, 96 dB/oct	Applied identically to OFF and ON base soundscapes to standardize low-frequency content and reduce interaction with 40 Hz layer.
Layering stage	40 Hz addition point	Added after HPF	Ensures the 40 Hz component itself is not attenuated by the HPF.
Level verification (post-export)	Standalone 40 Hz tone	−31.3 LUFS (integrated); true peak ≈ −24 dBTP	From final rendered files (Table 2).
	Waves OFF/ON	OFF: −18.0 LUFS; ON: −17.9 LUFS (Δ + 0.1 LUFS)	From final rendered files (Table 2).
	Forest OFF/ON	OFF: −19.7 LUFS; ON: −19.4 LUFS (Δ + 0.3 LUFS)	From final rendered files (Table 2).
Quality control	Clipping/DC offset	No clipping; negligible DC offset	From Table 2.
Exposure structure	Playback/ silence cycle	50 s playback + 10 s silence	7 cycles per condition (~7 min per condition).
	Washout	10 min between conditions	Within-session crossover.
Playback equipment	Earphones	Wired in-ear earphones (Sony XBA-A2)	Used for all sessions.
Playback control	Calibration	OS/player volume preset and held constant; SPL not individually calibrated	Perceived loudness may vary across individuals.

### Appendix A.2. COREQ Checklist

To enhance transparency and completeness of qualitative reporting, we provide the completed COREQ (Consolidated Criteria for Reporting Qualitative Research) checklist and indicate where each item is addressed in the manuscript.

**Table A2:** COREQ (32-item) checklist and manuscript location.

No.	Item (COREQ)	Where Reported (Section/Table/Figure)
	Domain 1: Research team and reflexivity	
1	Interviewer/facilitator	Section 2.8
2	Credentials	Title page; Section 2.8
3	Occupation	Title page (affiliations); Section 2.8
4	Gender	Section 2.8
5	Experience and training	Section 2.8
6	Relationship established	Section 2.8
7	Participant knowledge of the interviewer	Section 2.8
8	Interviewer characteristics/reflexivity	Section 2.8
	Domain 2: Study design	
9	Methodological orientation and theory	Section 2.1; Section 2.8 (inductive thematic analysis; TFA/Proctor sensitizing frameworks)
10	Sampling	Section 2.3
11	Method of approach	Section 2.3
12	Sample size	Section 2.3
13	Non-participation	Section 2.3
14	Setting of data collection	Section 2.5; Section 2.6
15	Presence of non-participants	Section 2.6 (no non-participants present)
16	Description of sample	Section 2.3; Table 1
17	Interview guide	Section 2.6; Table 3
18	Repeat interviews	Section 2.8 (not conducted)
19	Audio/visual recording	Section 2.2; Section 2.8
20	Field notes	Section 2.8
21	Duration	Section 2.6 (≈30 min per participant)
22	Data saturation	Section 2.8 (no claim of saturation)
23	Transcripts returned to participants	Section 2.8 (not returned)
	Domain 3: Analysis and findings	
24	Number of data coders	Section 2.8
25	Description of the coding tree	Section 2.8 (codebook development; audit trail)
26	Derivation of themes	Section 2.8
27	Software	Section 2.8 (spreadsheet-based coding matrices)
28	Participant checking (member checking)	Section 2.8 (not performed)
29	Quotations presented	Results; Table 5
30	Data and findings consistent	Results Section 3.2, Section 3.3, Section 3.4, Section 3.5, Section 3.6, Section 3.7 and Section 3.8; Table 5
31	Clarity of major themes	Results Section 3.2, Section 3.3, Section 3.4, Section 3.5, Section 3.6, Section 3.7 and Section 3.8; Table 5
32	Clarity of minor themes/diverse cases	Results narrative; Table 5 notes
